# Mithramycin selectively attenuates DNA-damage-induced neuronal cell death

**DOI:** 10.1038/s41419-020-02774-6

**Published:** 2020-07-27

**Authors:** Oleg Makarevich, Boris Sabirzhanov, Taryn G. Aubrecht, Ethan P. Glaser, Brian M. Polster, Rebecca J. Henry, Alan I. Faden, Bogdan A. Stoica

**Affiliations:** Department of Anesthesiology and Shock, Trauma and Anesthesiology Research (STAR) Center, University of Maryland School of Medicine, Baltimore, MD 21201 USA

**Keywords:** Transcriptional regulatory elements, Cell death in the nervous system, Molecular neuroscience

## Abstract

DNA damage triggers cell death mechanisms contributing to neuronal loss and cognitive decline in neurological disorders, including traumatic brain injury (TBI), and as a side effect of chemotherapy. Mithramycin, which competitively targets chromatin-binding sites of specificity protein 1 (Sp1), was used to examine previously unexplored neuronal cell death regulatory mechanisms via rat primary neurons in vitro and after TBI in mice (males). In primary neurons exposed to DNA-damage-inducing chemotherapy drugs in vitro we showed that DNA breaks sequentially initiate DNA-damage responses, including phosphorylation of ATM, H_2_AX and tumor protein 53 (p53), transcriptional activation of pro-apoptotic BH3-only proteins, and mitochondrial outer membrane permeabilization (MOMP), activating caspase-dependent and caspase-independent intrinsic apoptosis. Mithramycin was highly neuroprotective in DNA-damage-dependent neuronal cell death, inhibiting chemotherapeutic-induced cell death cascades downstream of ATM and p53 phosphorylation/activation but upstream of p53-induced expression of pro-apoptotic molecules. Mithramycin reduced neuronal upregulation of BH3-only proteins and mitochondrial dysfunction, attenuated caspase-3/7 activation and caspase substrates’ cleavage, and limited c-Jun activation. Chromatin immunoprecipitation indicated that mithramycin attenuates Sp1 binding to pro-apoptotic gene promoters without altering p53 binding suggesting it acts by removing cofactors required for p53 transactivation. In contrast, the DNA-damage-independent neuronal death models displayed caspase initiation in the absence of p53/BH3 activation and were not protected even when mithramycin reduced caspase activation. Interestingly, experimental TBI triggers a multiplicity of neuronal death mechanisms. Although markers of DNA-damage/p53-dependent intrinsic apoptosis are detected acutely in the injured cortex and are attenuated by mithramycin, these processes may play a reduced role in early neuronal death after TBI, as caspase-dependent mechanisms are repressed in mature neurons while other, mithramycin-resistant mechanisms are active. Our data suggest that Sp1 is required for p53-mediated transactivation of neuronal pro-apoptotic molecules and that mithramycin may attenuate neuronal cell death in conditions predominantly involving DNA-damage-induced p53-dependent intrinsic apoptosis.

## Introduction

Annually, over 4 million people in the US are impacted by traumatic brain injury (TBI)^1^ and almost 40% of patients demonstrate persistent functional decline^2^. TBI triggers multiple secondary injury processes leading to progressive neurodegeneration and related neurological deficits^3–5^. Increased production and accumulation of reactive oxygen species after TBI is a key secondary injury mechanism and can result in significant DNA damage and subsequent apoptosis^6^. Neurological dysfunctions are also a common side effect of cancer therapy with a substantial subset of patients suffering from chemotherapy-related cognitive impairment or “chemobrain”^7^. Drug-induced DNA damage and secondary central neurotoxicity may play a key role in cognitive impairments after chemotherapy^8^.

The intrinsic apoptosis pathway initiated by DNA damage^9^ involves p53 phosphorylation at serine 15^10,11^, followed by p53-dependent transcriptional activation of pro-apoptotic Bcl-2 family members^12,13^. These changes cause mitochondrial release^14,15^ of pro-apoptotic molecules such as cytochrome c (CytC) and apoptosis-inducing factor (AIF), leading to caspase-dependent and caspase-independent intrinsic apoptosis, respectively^10,15–17^. Experimental TBI models have also been associated with the production of single- and double-strand breaks in DNA and the resulting p53 activation contributes to neuronal loss and associated neurological deficits^18,19^. Accordingly, various studies have shown that p53 inhibition is neuroprotective^10,16,20–22^. However, p53 has also been shown to induce the expression of molecules required for neurite outgrowth and axonal regeneration and may promote regenerative responses following central nervous system injuries^23^. Thus, it is essential to separate maladaptive responses contributing to the neuropathology from adaptive responses enabling neuronal survival^24^. Total inhibition of p53 activities may be deleterious^25^, and an effective long-term strategy requires a more selective modulation of p53’s transcriptional effects.

Specificity protein 1 (Sp1) may regulate p53’s transcriptional profile and ability to promote apoptosis after DNA damage^26,27^. Furthermore, Sp1 can synergistically transactivate p53-binding promoters and may be necessary for p53-induced promoter transactivation^28^.

To examine the role of Sp1 in DNA-damage-induced neuronal apoptosis, a p53-transcription-dependent pathway, we utilized mithramycin, a drug that binds G–C-rich DNA to compete with Sp1 chromatin binding^29^ and has neuroprotective effects in several models involving p53 activation, including ischemic injury^29^, Huntington’s disease^30^, and spinal cord injury^31^. Pivotal studies by Ratan et al. showed that mithramycin inhibits apoptosis in models of DNA damage and oxidative stress in vitro^32,33^. However, no previous studies have comprehensively probed mithramycin’s mechanisms of action and examined its effects in DNA-damage-dependent and -independent neuronal intrinsic apoptosis models in vitro as well as after experimental TBI in vivo. We investigated mithramycin’s ability to attenuate specific cell death mechanisms activated in various models of neuronal death and to promote neuronal survival.

## Materials and methods

### Neuronal cell death and cell viability assays

Cell death was measured via LDH release (cytosolic LDH is released from dying cells due to membrane permeabilization) using the LDH-Glo^TM^ Cytotoxicity Assay (Cat. #J2380 Promega, Madison, WI) by combining 10 μL of media from a 96-well plate well with 10 μL of detection enzyme and reductase substrate, premixed just before assaying in the proportion recommended in the protocol and then diluted 1:10 in LDH Storage Buffer (also prepared as recommended in the protocol)^34^. Luminescence was measured after 1-h incubation in the dark in a BioTek Synergy HT Plate Reader using Gen5^TM^ software.

Calcein AM assay (Cat. #ALX-610-026, Enzo Life Sciences, Farmingdale, NY) was used to measure cell viability (calcein AM is retained in the cytosol of viable cells due to membrane integrity). Briefly, 1 mM stock of reagent in DMSO was added to pre-warmed Locke’s buffer (154 mM NaCl, 5.6 mM KCl, 3.6 mM NaHCO_3_, 5.6 mM glucose, 2.3 mM CaCl_2_, 5 mM HEPES, 1.2 mM MgCl_2_, pH 7.3) to a final concentration of 5 μM. Media was aspirated from 96-well plates and 100 μL of 5 μM calcein AM in Locke’s buffer was added to each well. Plates were incubated at 37 °C for 30 min after which fluorescent signal (excitation: 485 nm, emission: 528 nm) was read in a BioTek Synergy HT Plate Reader using Gen5^TM^ software.

### Primary cortical neuronal cultures

Embryonal rat cortical neurons (RCN) were derived as previously described from rat E15-16 cortices^35^. For each experiment, cortices were obtained from all embryos (mixed, unknown sex) of a single pregnant Sprague-Dawley® dam (Envigo). After dissociation, cells were seeded onto 96-well, 12-well, 60 mm, 100 mm cell culture plates (Corning, Corning, NY) or XF24 cell culture microplates (Agilent, Santa Clara, CA) and maintained in serum-free conditions using the B27 supplement (ThermoFisher, Waltham, MA) as described previously^35^. We previously showed these cultures are >90% neuronal^36^.

Etoposide (Cat. #BML-GR307, Enzo Life Sciences, Farmingdale, NY), staurosporine (Cat. #ALX-380-014, Enzo Life Sciences, Farmingdale, NY), camptothecin (Cat. #ALX-350-015, Enzo Life Sciences, Farmingdale, NY), C_2_-ceramide (Cat. #BML-SL100, Enzo Life Sciences, Farmingdale, NY), doxorubicin (Cat. #CST-5927, Cell Signaling Technologies, Danvers, MA), and mithramycin (Cat. #11434, Cayman Chemical Company, Ann Arbor, MI) were used to treat 7 days in vitro (DIV) cells at concentrations and for times indicated elsewhere.

### Mitochondrial respiration measurement

After 6 h treatment in a XF24 cell culture microplate (Agilent, Santa Clara, CA), mitochondrial oxygen consumption rate (OCR) was measured by utilizing an Agilent Seahorse XF Analyzer as previously described^37,38^. Briefly, after 6 h treatment of RCN with etoposide +/− mithramycin, 0.5 µg/ml oligomycin, 200 µM dinitrophenol, 10 mM pyruvate, and 1 µM antimycin A were sequentially added to identify maximum and spare respiratory capacities. Pyruvate was added to ensure that endogenous substrate was not the limiting factor in maximal respiration measurements. Treatment length was chosen because time course analysis of LDH release (data not shown) indicated little to no cell death occurring 6 h after etoposide treatment.

### RNA isolation and quantitative qPCR

Total RNA was isolated using either the miRNeasy kit (QIAGEN, Hilden, Germany) or the Zymo Research Direct-zol RNA kits (Zymo Research, Irvine, CA) according to the manufacturers’ protocols. Either the Verso cDNA Synthesis kit (Cat. #AB1453B, ThermoFisher, Waltham, MA) or the High-Capacity cDNA Reverse Transcription kit (Cat. #4368813, ThermoFisher, Waltham, MA) was used to synthesize cDNA from purified RNA based on the manufacturer’s protocol. Quantitative real-time PCR was performed using TaqMan Universal Master Mix II (Applied Biosystems, Foster City, CA) with 50 ng cDNA per sample, in duplicate, using the following TaqMan^TM^ primers: Bbc3/PUMA: Rn00597992_m1; Pmaip1/Noxa: Rn01494552_m1; Cdkn1a/p21: Rn01427989_s1; Akt1: Rn00583646_m1; Ang-1: Rn01504818_m1.

Reactions were amplified and quantified via an Applied Biosytems QuantStudio 5 and its corresponding software (Applied Biosystems, Foster City, CA). The PCR profile consisted of one cycle of 50 °C for 2 min and 95 °C for 10 min, followed by 40 cycles of 95 °C for 15 s and 60 °C for 1 min. Gene expression was normalized to GAPDH and the relative quantities of mRNA calculated using the 2^−ddCt^ method as described previously^39^.

### MicroRNA reverse transcription and qPCR

100 ng/sample of total RNA was isolated as described above and reverse transcribed using TaqMan^TM^ MicroRNA Reverse Transcription Kit (Cat #4366596, ThermoFisher, Waltham, MA) and TaqMan^TM^ primers. Equal volumes of each sample were loaded in duplicate for qPCR using TaqMan^TM^ Advanced miRNA assays and the following primers: miR-711: Cat. #241136_mat; miR-23a: Cat. #000319.

Reactions were amplified and quantified via an Applied Biosytems QuantStudio 5 and its corresponding software (Applied Biosystems, Foster City, CA). The PCR profile consisted of one cycle of 50 °C for 2 min and 95 °C for 10 min, followed by 40 cycles of 95 °C for 15 s and 60 °C for 1 min. Gene expression was normalized to U6 small nucleolar RNA and the relative quantities of miRNA calculated using the 2^−ddCt^ method as described previously^39^.

### Cell lysate preparation and western blot

After 24 h (or less) treatment of RCN with cell death inducer +/− mithramycin, cells were quickly scraped from the dish and added to cold 1X PBS. The mixture was centrifuged at 1000 × *g* to pellet cells and supernatant was removed. RIPA buffer (Cat #R3792, Teknova, Hollister, CA) with Protease Inhibitor and Phosphatase Inhibitor (2,3) cocktails (Sigma-Aldrich, St. Louis, MO) was added to the pellet and complete lysis was ensured by incubating the lysate at 4 °C with rocking for 30 min and vortexing thoroughly every 10 min during the incubation. To ensure appropriate comparisons between samples, we took the two-pronged approach of both loading equal amounts of protein and normalizing to an appropriate housekeeping protein. Protein concentration was measured using Pierce^TM^ BCA Protein Assay Kit (ThermoFisher, Waltham, MA) according to the manufacturer’s instructions. Equal amounts of protein were loaded onto 4–20% Criterion^TM^ TGX^TM^ Precast Midi Protein Gels (Bio-Rad, Hercules, CA) and electrophoresis was performed. Proteins were transferred to 0.2 µm nitrocellulose membranes using the Trans-Blot® Turbo^TM^ (Bio-Rad, Hercules, CA). Membranes were washed, incubated with primary and secondary antibodies (see antibody list), and complexes were visualized using SuperSignal^TM^ West Dura Extended Duration Susbtrate (ThermoFisher, Waltham, MA). To assess proteins with sufficiently separate molecular weights, membranes were cut into sections and probed separately using supplier-validated antibodies.

Chemiluminescence was captured on a ChemiDoc^TM^ Touch Imaging System (Bio-Rad, Hercules, CA) and protein bands were quantified by densitometric analysis using ImageLab software (Bio-Rad, Hercules, CA). Images were acquired under non-saturating conditions and were normalized to an endogenous control for each sample (arbitrary units). When membranes were sectioned for separate analysis, each section was normalized to the endogenous control from the same membrane. All quantifications are presented after normalization.

### Subcellular fractionation

Subcellular fractionation was performed as described previously^36^. Briefly, RCN were harvested and washed in ice-cold phosphate-buffered saline. The cell suspension was centrifuged at 500 × *g* for 15 min at 4 °C. The cell pellet was resuspended for 10 min on ice in digitonin lysis buffer (20 mM HEPES, pH 7.4, 80 mM KCl, 1 mM EDTA, 1 mM EGTA, 1 mM DTT, 250 mM sucrose, 200 μg/mL digitonin, and protease inhibitor and phosphatase inhibitor (2,3) cocktails (Sigma-Aldrich, St. Louis, MO). Cells were passaged 20 times through a 22G needle. The lysate was centrifuged at 1000 × *g* for 5 min at 4 °C to pellet the nuclei. The supernatant was transferred to a new tube and centrifuged again at 12,000 × *g* for 10 min at 4 °C to pellet the mitochondria. The resulting supernatant, representing the cytosolic fraction, was recovered. Nuclear and mitochondrial lysates were prepared in RIPA buffer (Cat #R3792, Teknova, Hollister, CA) with protease inhibitor and phosphatase inhibitor (2,3) cocktails (Sigma-Aldrich). All steps were performed on ice. Pooled nuclear, cysotolic and total lysates were probed via electrophoresis and western blot for COX IV to identify mitochondrial content and Lamin to identify nuclear content to verify fractionation procedure.

### Chromatin immunoprecipitation (ChIP)

ChIP assay was performed as previously described^37^ using a kit from Epigentek (Farmindale, NY), with 2 μg of antibodies to p53 or 4 μg of antibodies to Sp1 per sample. Briefly, after cross-linking using 1% formalehyde in PBS, cells were lysed and chromatin sheared to generate fragments from 200 to 600 bp using a Bioruptor sonicator (Diagenode). After this, immunoprecipitation was performed. The following binding sites were analyzed using the following primer sequences (all nt locations from Rnor_6.0 chromosome 18). Primers were ordered from Integrated DNA Technologies (Coralville, Iowa).

Primers:

5′-CTTCCCTCCCACCTTCGTTT-3′ (62,174,414 –62,174,434 nt)

5′-GCCGGCTCTCGGGTTTTAT-3′ (62,174,653–62,174,872 nt)

p53 site: 5′-CGGCTTGCCCCGGCAAGTTG-3′ (62,174,513–62,174,533 nt)

Sp1 site: 5′-TTCGAAGGGGCGGGG-3′ (62,174,589–62,174,604 nt).

Rat negative control primer set 1 (Active Motif) was also used to amplify a fragment of a gene desert on rat chromosome 3 as a negative control. Normal rabbit IgG (#3900 Cell Signaling Technologies, Danvers, MA) and anti-RNA polymerase II antibody (Epigentek, Farmingdale, NY) were used as negative and positive controls to validate the primer set used on both IPs. The difference between the negative control IP and the average sample IP was 21.8-fold, while the difference between the positive control IP and the average sample IP was 3.33-fold. A dilution series was utilized to calculate the reaction efficiency, and it was found to be 77.1% for our primer pair. Relative expression was calculated using the Pfaffl method^40^.

### Immunocytochemistry

For the phospho-c-Jun (S73) time course, RCN were treated with etoposide +/− mithramycin on DIV 7 in 24-well plates with coverslips. After 6, 12, or 24 h, cells were fixed for 10 min in 4% paraformaldehyde/PBS and co-stained with a 1:200 dilution of Cell Signaling’s phospho-c-Jun (S73) and a 1:400 dilution of Millipore’s Milli-Mark^TM^ Pan-Neuronal Marker in 10% goat serum (Gemini Bio-Products, West Sacramento, CA) overnight. Wells were incubated the next day with goat-derived secondary antibody (Life Technologies, Fisher Scientific, Hampton, NH), followed by 4′,6-diamidino-2-phenylindole (DAPI, Sigma-Aldrich, St. Louis, MO) (0.5 µg/mL in saline). Imaging was performed via a Leica SP5 II confocal microscope using a ×20 dry objective and ×63 oil-immersion objective. Settings were optimized to maximize signal intensity in controls without oversaturating signal in higher-intensity samples (etoposide-treated). Analysis was done using an ImageJ macro as described previously^37^ to generate an unbinned cumulative frequency distribution plotting intensity of phospho-c-Jun (S73) signal per cell.

For the phospho-p53 (S15) assessment at 6 h, RCN were treated with etoposide +/− mithramycin or Mithramycin alone on DIV 7 in 24-well plates with coverslips. After 6 h, cells were fixed and stained with Abcam’s phospho-p53 (S15) antibody. Imaging was performed via a Nikon Ti-E fluorescent microscope using a ×63 oil-immersion objective. Analysis was performed using Nikon’s NIS-Elements software to generate an unbinned cumulative frequency distribution plotting intensity of phospho-p53 (S15) signal per cell.

To compare cell death inducers, RCN were treated with doxorubicin, etoposide, C_2_-ceramide, staurosporine +/− mithramycin on DIV 7 in 24-well plates with coverslips. After 6 h, cells were fixed and co-stained as described above with a 1:200 dilution of Cell Signaling’s cleaved PARP (94885), cleaved Casp3 (9664) or phospho-c-Jun (S73) antibodies and Millipore’s Milli-Mark^TM^ antibody. Imaging was performed via a Nikon Ti-E fluorescent microscope using a ×63 oil-immersion objective. Analysis was performed via Nikon’s NIS-Elements software to generate an unbinned cumulative frequency distribution plotting intensity of phospho-c-Jun (S73) signal per cell. It was also used for identifying total cleaved PARP or cleaved Casp3 signal per field (normalized to the number of nuclei per field) for quantification of cleaved PARP and cleaved Casp3.

### Antibodies

Various antibodies from different vendors were used in this study.

Cell Signaling (Danvers, MA): PARP (9542); cleaved PARP [Asp214, 89 kDa fragment] (9545/94885); cleaved Casp3 [Asp175, 17 kDa fragment] (9661/9664); cleaved Casp7 [20 kDa fragment] (9492); phospho-H_2_AX (S139) (9718); phospho-p53 (S15) (12571); total p53 for ChIP (32532); PUMA (14570); AIF (4642); cytochrome c oxygenase IV (COX IV) (4884); Lamin A/C (4777); phospho-c-Jun (S63) (2361); phospho-c-Jun (S73) (3270); total c-Jun (9165); total c-Fos (2250). Millipore (Ontario, Canada): phospho-ATM(S1981) (05-740); Sp1 for ChIP (07-645); p21—mouse samples (188224); Milli-Mark^TM^ Pan-Neuronal Marker (MAB2300). BD Biosciences (San Jose, CA): p21—rat samples (556430). Sigma-Aldrich (St. Louis, MO): β-actin: A1978. Santa Cruz (Dallas, TX): cytochrome c (sc-13560). Enzo (Farmingdale, NY): α-Fodrin or αII-spectrin (a subunit of Fodrin, also known as “brain spectrin”)^41^: BML-FG6090. Abcam (Cambridge, MA): phospho-p53 (ab1431).

### Mice

Eight-week-old (20–25 g) male C57BL/6 mice were obtained from JAX (Jackson Laboratories, Bar Harbor, ME) for the in vivo experiments. Mice were maintained in a 12-h light/dark cycle with ad libitum access to food and water. All activities were in accordance with protocols approved by the University of Maryland School of Animal Care and Use Committee and complied with the Guide for the Care and Use of Laboratory Animals published by NIH (DHEW publication NIH 85-23-2985).

### Controlled cortical impact

We utilized a custom-designed CCI injury device^37,42,43^ consisting of a microprocessor-controlled pneumatic impactor with a 3.5 mm diameter tip. Mice were anesthetized with isofluorane (3–3.5% induction, 1.5% maintenance) in a 70% NO, 30% O_2_ gas mixture administered via nose mask. Animals were monitored during procedures to assess depth of anesthesia via respiration rate and pedal withdrawal reflexes. The surgical site was clipped, then the head mounted in a stereotaxic frame and the injury site cleaned with betadine (Professional Disposables, Orangebury, NY) and ethanol scrubs (Fisher Scientific, Hampton, NH). Mice received puralube vet ointment eye lubrication (Dechra Veterinary Products, Overland Park, KS). A 10-mm midline incision was made over the skull, the skin and fascia were reflected, and a 5 mm craniotomy was made on the central aspect of the left parietal bone^37,44^. The impactor tip of the injury device was extended to its full stroke distance (44 mm) and positioned to the surface of the exposed dura, then reset to impact the cortical surface. Moderate injury was performed using impactor velocity of 6 m/s and deformation depth of 2 mm, as previously described^45^. The incision was then closed via 6 mm nylon surgical sutures (Unify® Premium+, AD Surgical, Sunnyvale, CA) and anesthesia was terminated. Mice were randomly assigned to CCI or sham groups. Sham mice underwent only anesthesia and incision (with no craniotomy).

### Drug administration

Following injury, mice received an intracerebroventricular (icv) injection of 0.3 mM mithramycin in artificial CSF (#59-7316, Harvard Apparatus, Holliston, MA) or equal volume of vehicle (artificial CSF + DMSO). Solutions were prepared on the day of surgery. The injection was performed immediately after injury via 30-gauge needle attached to Hamilton syringe (Hamilton, Reno, NV) into the left ventricle (anterior posterior (AP): −0.5, medial lateral (ML): −1.0, dorsal ventral (DV): −2.0 from bregma) at a rate of 0.5 µL/min with a final volume of 5 µL (1.5 nmoles) infused over 10 min. This dose was based on prior work utilizing CR8^37^, and comparisons of the two compounds’ neuroprotective effects in vitro. CCI mice were randomly assigned to an icv treatment group with a final total of 7 (CCI + saline) or 8 (CCI + mithramycin) animals per group. At 24 h post-injury the animals were euthanized and the injured cortex was analyzed.

### Experimental design and statistical analysis

For in vitro LDH, calcein, qPCR, and western blot assays, at least three separate wells of primary RCN seeded on day 0 from the same primary culture were used for any given assay. These separately cultured and treated neurons isolated from the same pool of embryos were run on the same gel/assay plate and quantified as indicated elsewhere. Each set of experiments (except ChIP) involving etoposide +/− mithramycin was repeated at least twice in an equivalent manner with another pool of embryos from a different pregnant dam and showed consistent results. Experiments with other cell death inducers besides etoposide were repeated at least once. For mitochondrial function analysis, each plate (*n* = 4) came from a separate pool of embryos, and each contained separately cultured and treated neurons (different wells, each seeded with the same number of neurons) isolated from the same pool of embryos.

All immunofluorescent analysis was done on multiple fields from a single coverslip for each treatment and each timepoint. These coverslips came from separately cultured and treated neurons (different wells) isolated from a single pool of embryos. The experiments were repeated as described in the paragraph above and showed consistent results.

Statistical analysis was performed using GraphPad Prism 8 (La Jolla, CA). For mitochondrial function tests, we used a one-way repeated-measures ANOVA with Tukey’s post hoc tests. LDH, Calcein, ChIP, qPCR and western blot assays were all analyzed via one-way ANOVA with Tukey’s post hoc tests. Immunocytochemically stained cells were analyzed using the Kruskal–Wallis test followed by Dunn’s post hoc analysis for phospho-P53 and phospho-c-Jun (S73) and with one-way ANOVA with Tukey’s post hoc tests for cleaved PARP and cleaved Casp3 analyses. All data analyzed with one-way ANOVA with Tukey’s post hoc tests met the normality assumption by the Shapiro–Wilk test. Otherwise, the data were analyzed using the Kruskal–Wallis test, followed by Dunn’s post hoc tests. When appropriate, we tested for unequal variance by the Brown–Forsythe test and if unequal, we utilized the Brown–Forsythe ANOVA test followed by Dunnett’s T3 multiple comparisons test.

## Results

### Mithramycin’s neuroprotective effects are cell death model-specific

**Fig. 1:**
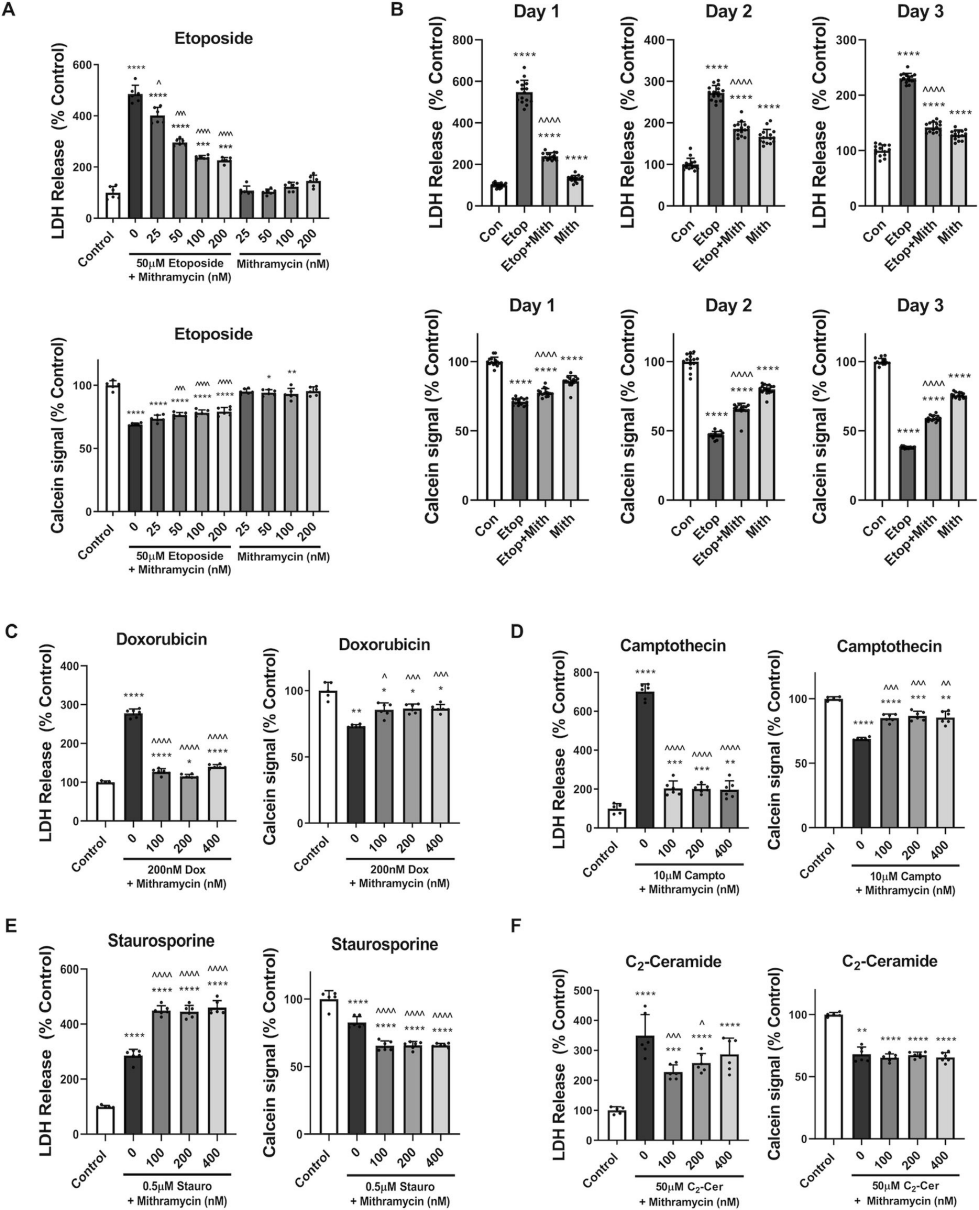
Mithramycin’s neuroprotective effects are cell death model-specific. **a** RCN were treated with varying doses of mithramycin (Mith) +/−50 µM etoposide (Etop). LDH release and calcein signal were measured after 24 h of treatment. **b** RCN were treated with 50 µM etoposide and/or 200nM mithramycin. Treatment media was replaced with conditioned media at 24h. LDH release and calcein signal were measured 24, 48, and 72 h after treatment. **c**–**f** RCN were treated with 200 nM doxorubicin (Dox) (**c**), 10µM camptothecin (Campto) (**d**), 0.5 µM staurosporine (Stauro) (**e**), or 50 µM C_2_-ceramide (C_2_-Cer) (**f**) with or without varying doses of mithramycin. *n* = 5+/group for all groups. Data represent mean± SD. Significance assigned based on one-way ANOVA and Tukey post hoc test or Brown–Forsythe ANOVA and Dunnett’s T3 multiple comparisons test [**a** LDH, **b** LDH Day 1, calcein Day 3, **c**, **d**, **f** Calcein]; *p < 0.05, **p < 0.01, ***p < 0.001, ****p < 0.0001 vs control; ^*p* < 0.05, ^^*p* < 0.01, ^^^*p* < 0.001, ^^^^*p*  0.0001 vs etoposide alone.

Etoposide (DNA-damage inducer; 50 µM) treatment (24 h) resulted in significant rat cortical neuron (RCN) death, as shown by increased LDH release (indicator of dying cells) and decreased calcein signal (indicator of surviving cells) (Fig. 1a). Mithramycin co-treatment had significant, dose-dependent neuroprotective effects in both measures (Fig. 1a). We subsequently used 200 nM mithramycin, except when indicated.

To characterize mithramycin’s long-term neuroprotective effects, RCN were treated for 24 h followed by media replacement with RCN-conditioned media (no etoposide or mithramycin) and further incubation until 48 or 72 h. RCN treated with etoposide showed increased cell death (higher LDH) and progressively decreased survival (lower calcein) at 24, 48, and 72 h after treatment (Fig. 1b). Decreased LDH signal levels vs. controls at 48 and 72 h are due to media replacement after 24 h. Mithramycin co-treatment had a long-term neuroprotective effect in both measures at all time points. Mithramycin alone resulted in a minor decrease in neuronal viability, especially at later time points (Fig. 1a, b).

We also examined mithramycin’s neuroprotective effects in additional DNA-damage-dependent (campthothecin/doxorubicin) as well as DNA-damage-independent (staurosporine/C_2_-ceramide) cell death models. Doxorubicin (200 nM) (Fig. 1c) or camptothecin (10 µM) (Fig. 1d) caused significant RCN death by both LDH and calcein measures. Mithramycin co-treatment was neuroprotective at all tested doses.

Staurosporine (0.5 µM) (Fig. 1e) or C_2_-ceramide (50 µM) (Fig. 1f) caused RCN death by both LDH and calcein measures. Mithramycin co-treatment had a minor neuroprotective effect in the C_2_-ceramide model as indicated by the decrease in LDH release; no effect was detected on calcein signal. Interestingly, mithramycin co-treatment resulted in increased RCN cell death in the staurosporine model by both measures.

### Mithramycin attenuates etoposide-induced apoptotic pathways downstream of p53 activation

**Fig. 2:**
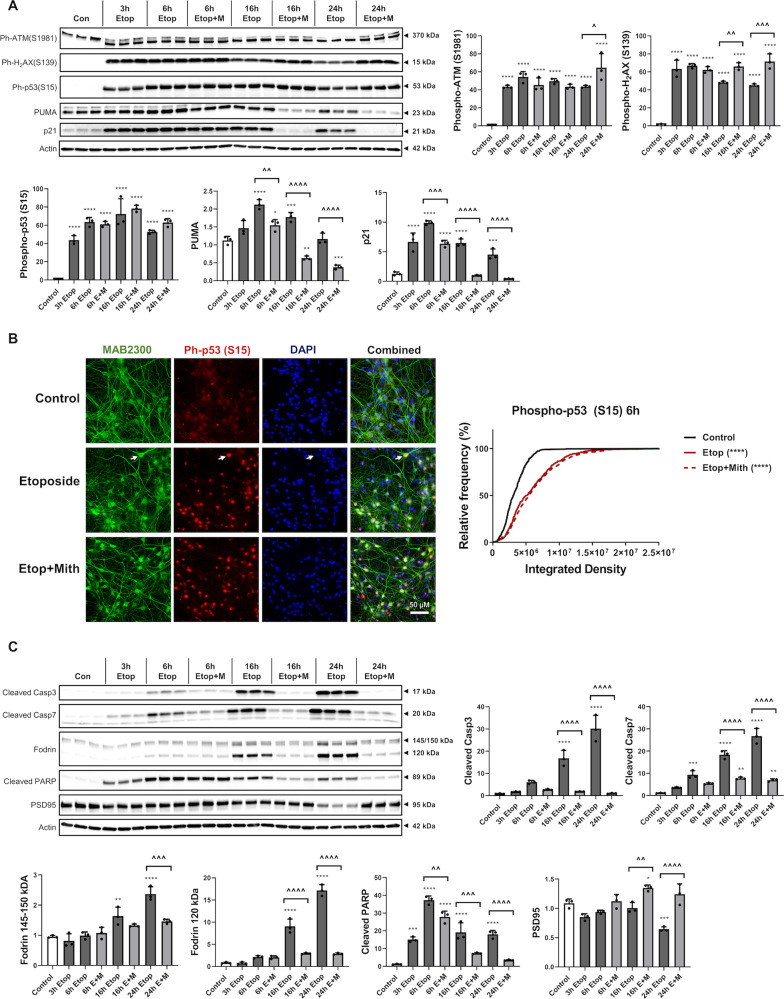
Mithramycin attenuates etoposide-induced apoptotic pathways downstream of p53 activation. **a**, **c** RCN were treated with 50µM etoposide +/−200 nM mithramycin. After 3, 6, 16, or 24 h, cells were harvested. Equal amounts of whole-cell lysates were loaded onto an SDS-polyacrylamide gel and after electrophoretic separation and transfer to a membrane were incubated with antibodies against DNA-damage markers: phospho-ATM (S1981) and γH_2_AX, or phospho-p53 (S15) and its downstream targets: PUMA and p21 (**a**). Other membranes were incubated with antibodies against cleaved/activated caspase-3 (Casp3) and caspase-7 (Casp7), their substrates α-Fodrin and cleaved PARP, as well as PSD95 (**c**). Protein levels (of bands indicated by arrows) were quantified by densitometry, normalized to appropriate β-actin signal and are presented as normalized fold change compared with control levels. Representative actin blots are shown here. *n* = 3/group for all groups. Data represent mean ±SD. Significance assigned based on one-way ANOVA and Tukey post hoc test; *p < 0.05, **p < 0.01, ***p < 0.001, ****p < 0.0001 vs control; ^*p* < 0.05, ^^*p* < 0.01, ^^^*p* < 0.001, ^^^^*p* < 0.0001 vs etoposide alone. **b** Phospho-p53 (S15) levels were also analyzed by immunocytochemistry after 6h. A representative set of  × 63 images is shown here, white arrows indicate sample neuron co-localized with ph-p53 (S15). Quantification of cells was done across at least 6 fields per treatment. Data are presented as an unbinned cumulative frequency distribution. Significance assigned based on Kruskal–Wallis test followed by Dunn’s post hoc analysis; ****p < 0.0001 vs control.

Etoposide induced DNA-damage markers, phosphorylated ataxia-telangiectasia, mutated (ATM) (S1981) and phosphorylated H2A histone family member X (S139) (γH_2_AX) at all time points (western blot) (Fig. 2a). Co-treatment with mithramycin did not significantly change the levels of either marker, except a modest increase at later time points that may reflect increased neuronal survival (Fig. 2a). Lack of mithramycin effect on γH_2_AX was also confirmed by immunocytochemistry (data not shown). Quantitative analysis of phospho-p53 immunocytochemistry and western blotting (Fig. 2a, b) indicated that etoposide increased levels of activated p53 [mean rank diff. = 355.6 in Fig. 2b]. Mithramycin co-treatment had no significant effects on etoposide-induced increases in phospho-p53 levels (western blot) (Fig. 2a) or population distribution (immunocytochemistry) (Fig. 2b).

Etoposide also resulted in elevations of pro-apoptotic molecules downstream of p53, including p53-upregulated mediator of apoptosis (PUMA) and cyclin-dependent kinase inhibitor 1 (cdkn1a/p21), that were significantly attenuated via mithramycin co-treatment (western blot) (Fig. 2a).

Etoposide-treated RCN showed increased execution-stage apoptosis markers including activated (cleaved fragment) caspase-3 and caspase-7 as well as increased cleavage of substrates, Fodrin (120 kDa) and PARP (89 kDa) (Fig. 2c). The increase in Fodrin’s calpain/caspase-dependent cleavage fragments (145/150 kDa) was more modest. Mithramycin co-treatment significantly attenuated all described caspase activation markers/cleavage products as well as the etoposide-induced decrease in synaptic marker PSD95, a general indicator of neuronal degeneration/loss (Fig. 2c).

### Mithramycin reduces etoposide-induced mitochondrial damage and attenuates p53-dependent transcription

**Fig. 3:**
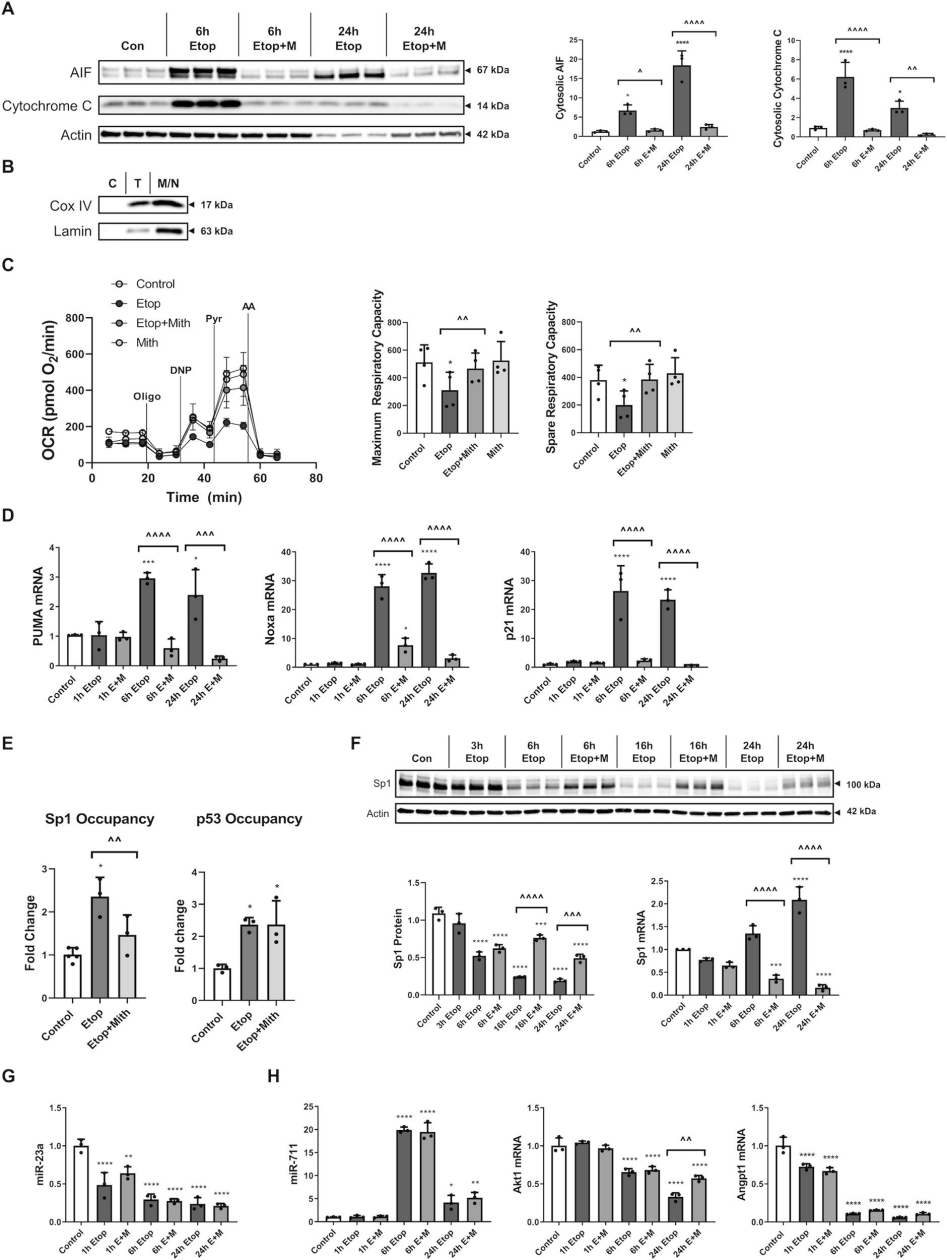
Mithramycin reduces etoposide-induced mitochondrial damage and attenuates p53-dependent transcription. RCNs were treated with 50 µM etoposide +/−200 nM Mithramycin for all panels. **a** The cytosolic fraction was collected after 6h and 24 h. Controls were collected at 24h. Equal amounts of fraction lysates were loaded onto an SDS-polyacrylamide gel and after electrophoretic separation and transfer to a membrane were incubated with antibodies against AIF and cytochrome C proteins. Protein levels (of bands indicated by arrows) were quantified by densitometry, normalized to β-actin signal and are presented as normalized fold change compared with control levels. *n* = 3/group for all groups. **b** Cytoplasmic (C), total (T), and nuclear/mitochondrial (N/M) fractions were pooled and probed for cytochrome c oxidase IV (COX IV) and Lamin to confirm purity of cytoplasmic fraction from mitochondria and nuclei, respectively. **c** 6h after treatments, cellular respiration was measured using a Seahorse XF24 Extracellular Flux Analyzer, and a representative measurement is shown here. Sequential addition of oligomycin, DNP, pyruvate and antimycin A was utilized to identify maximum and spare respiratory capacity (**c**). Each group contains *n* = 4 averages from separate experiments on different days, each experiment except one contained *n* = 4+ separately cultured wells of neurons per group (that experiment contained wells that were eliminated due to no significant increase in OCR over baseline indicating a failed injection/port, *n* = 3+/group for that experiment). **d**, **f**–**h** After 1, 6, or 24h, cells were harvested. Equal amounts of purified RNA were converted into cDNA. Equal volumes of cDNA were loaded for qPCR. mRNA/miRNA levels were normalized via U6/GAPDH (respectively), quantified using the ddCt method and are presented as fold change compared with control levels. *n* = 3/group for all groups. **e** Chromatin Immunoprecipitation was done using p53 or Sp1 antibodies, and equal volumes of resulting DNA fragments were loaded for qPCR. Pulled-down DNA levels were normalized using ChIP Inputs, quantified using the 2^−ddCt^ method and are presented as fold change compared with control levels. **f** Electrophoresis and western blotting were performed for Sp1 protein. Protein levels were quantified by densitometry, normalized to β-actin signal and are presented as normalized fold change compared with control levels. *n* = 3/group for all groups. Data all represent mean±SD. Significance assigned based on one-way ANOVA and Tukey post hoc test; *p < 0.05, **p <0.01, ***p < 0.001, ****p < 0.0001 vs control; ^*p* < 0.05, ^^*p* < 0.01, ^^^*p* < 0.001, ^^^^*p* < 0.0001 vs etoposide alone.

Etoposide treatment of RCN caused early (6 h) mitochondrial outer membrane permeabilization (MOMP) as seen by cytosolic release of pro-apoptotic AIF and CytC (western blot) (Fig. 3a), while mithramycin co-treatment decreased release and thus showed preserved mitochondrial integrity (Fig. 3a). Standard normalizing proteins are degraded at late stages of apoptosis^46^, and both actin (Fig. 3a) and GAPDH (data not shown) showed a decline at 24 h in purified cytoplasmic fractions.

Etoposide treatment (6 h) also caused mitochondrial functional decline, demonstrated by significant reductions in maximum and spare respiratory capacities (Seahorse Analyzer) that were rescued by mithramycin co-treatment (Fig. 3b). We have previously shown no significant neuronal loss at the etoposide 6 h timepoint^37^.

Etoposide-treated RCN showed increased mRNA levels of PUMA, phorbol-12-myristate-13-acetate-induced protein 1 (Pmaip1/Noxa) and p21 (qPCR). These changes were substantially attenuated by Mithramycin co-treatment (Fig. 3d).

Chromatin Immunoprecipitation demonstrated that etoposide-treated RCN displayed increased Sp1- and p53 binding to the same Noxa promoter region vs. controls (Fig. 3e). Mithramycin co-treatment decreased only the Sp1 binding to this Noxa promoter region (Fig. 3e).

Etoposide increased Sp1 mRNA levels and mithramycin co-treatment attenuated these changes, likely because Sp1 activates its own transcription^47^. Protein levels of Sp1 show a divergent profile with an etoposide-induced decrease, likely due to early apoptotic degradation^48^, partially rescued by mithramycin co-treatment. Therefore, mithramycin’s effects are not driven by Sp1 protein loss (Fig. 3f).

Although etoposide decreased the levels of pro-survival miR-23a^35^ and increased levels of pro-apoptotic miR-711^49^ while decreasing the level of its target mRNAs^36,49^ vs. control, mithramycin co-treatment had no effect on these changes (qPCR) except a late (24 h) increase in Akt mRNA (Fig. 3g, h).

### Mithramycin attenuates etoposide-induced activation of the c-Jun injury response

**Fig. 4:**
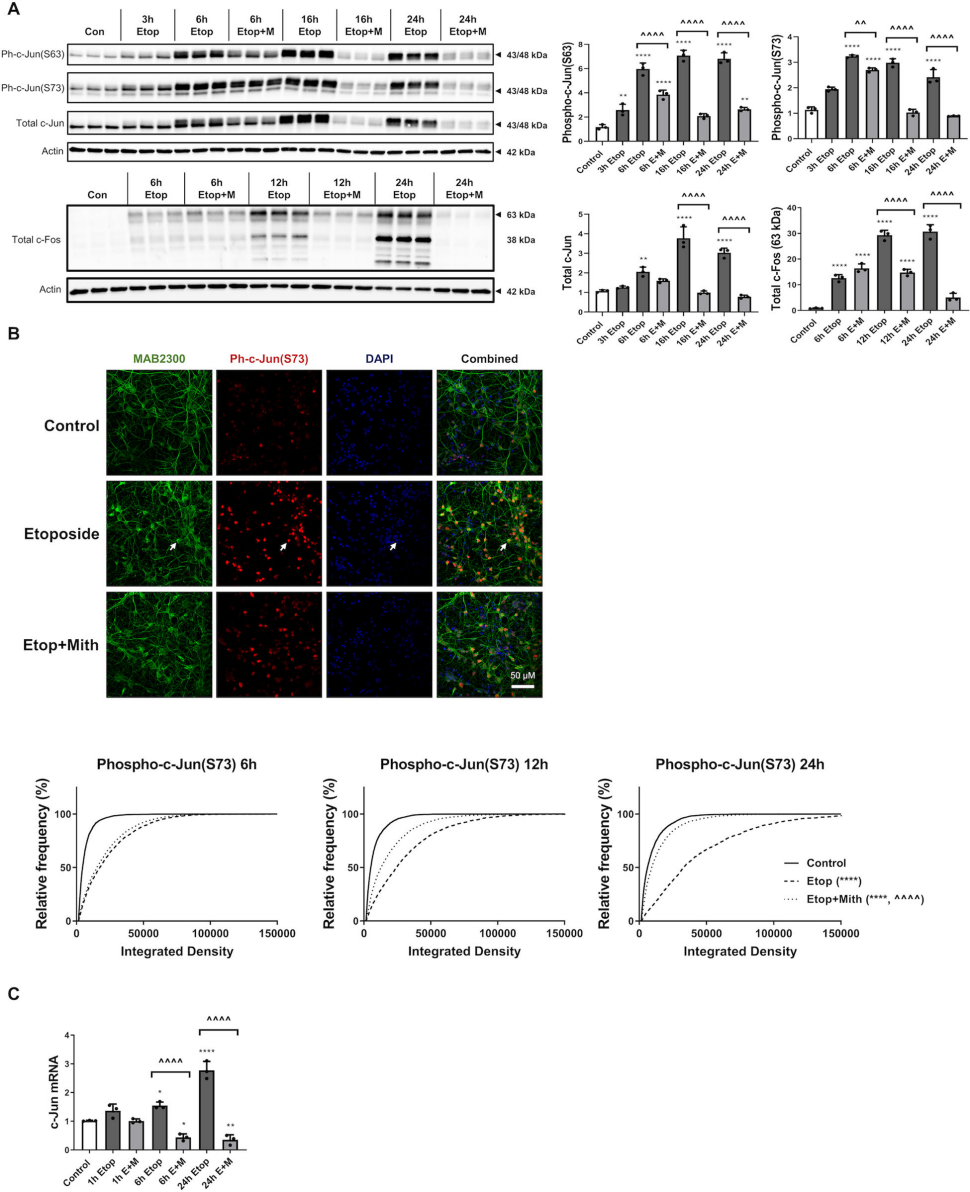
Mithramycin attenuates etoposide-induced activation of the c-Jun injury response. RCN were treated with 50 µM etoposide +/−200 nM mithramycin for all panels. **a** After 3, 6, 12, 16, or 24 h, cells were harvested. Equal amounts of whole-cell lysates were loaded onto an SDS-polyacrylamide gel and after electrophoretic separation and transfer to a membrane were incubated with antibodies against phospho-c-Jun (S63), phospho-c-Jun (S73), total c-Jun, and total c-Fos proteins. Protein levels (of bands indicated by arrows) were quantified by densitometry, normalized to appropriate β-actin signal and are presented as normalized fold change compared with control levels. Representative actin blot is shown here. *n* = 3/groups for all groups. Data all represent mean ± SD. Significance assigned based on one-way ANOVA and Tukey post hoc test; **p < 0.01, ****p < 0.0001 vs control; ^^*p* < 0.01, ^^^^*p* < 0.0001 vs etoposide alone. **b** RCN were fixed with formaldehyde 6, 12, or 24h after treatment and stained with antibodies for neuronal markers (MAB2300, Neuro-Chrom™ Pan-Neuronal Marker), ph-c-Jun (S73), and DAPI for fluorescent imaging. Representative images are from ×63 magnification at 12 h, white arrows indicate sample neuron co-localized with ph-c-Jun (S73). At least four fields at ×20 magnification were quantified for each treatment as described in materials and methods. Data were graphed as an unbinned cumulative frequency distribution. Using Kruskall–Wallis test, followed by Dunn’s post hoc analysis, all groups at all time points had significantly different distributions, ****p < 0.0001 vs control, ^^^^*p* < 0.0001 vs etoposide alone. **c** After 1, 6, or 24h, cells were harvested. Equal amounts of purified RNA were converted into cDNA. Equal volumes of cDNA were loaded for qPCR. mRNA levels were normalized via GAPDH, quantified using the 2^−ddCt^ method and are presented as fold change compared with control levels. *n* = 3/group for all groups. Data all represent mean±SD. Significance assigned based on one-way ANOVA and Tukey post hoc test; *p < 0.05, **p < 0.01, ****p < 0.0001 vs control; ^^^^*p* < 0.0001 vs etoposide alone.

RCN treated with etoposide showed c-Jun activation, including significantly increased levels of phospho-c-Jun (Ser63), phospho-c-Jun (Ser73) and total c-Jun (western blot) (Fig. 4a). Etoposide treatment also increased c-Fos protein levels. Mithramycin co-treatment significantly reduced all four markers (western blot) (Fig. 4a). Multiple close c-Jun bands suggest extensive post-translational modification, including phosphorylation, causing gel mobility shifts. In addition, the c-Jun antibody recognizes both a 43 kDa and 48 kDa form. In in vitro samples, we quantified these bands together as they were both present and regulated in parallel.

Quantitative analysis of phospho-c-Jun (S73) immunocytochemistry showed a progressive increase in signal intensity of phospho-c-Jun (S73)-positive neurons in etoposide-treated RCN (Fig. 6b). Mithramycin co-treatment reduced these changes, pushing the population distribution curve to the left (Fig. 4b), with a mean rank difference that progressively increased and was 288 at 6 h, 1227 at 12 h, and 2163 at 24 h (Fig. 4b).

Etoposide increased c-Jun mRNA levels (6 and 24 h); these changes were attenuated by mithramycin co-treatment to below control levels (Fig. 4c).

### Mithramycin’s attenuation of apoptotic mechanisms in neurons is cell death model-specific

**Fig. 5:**
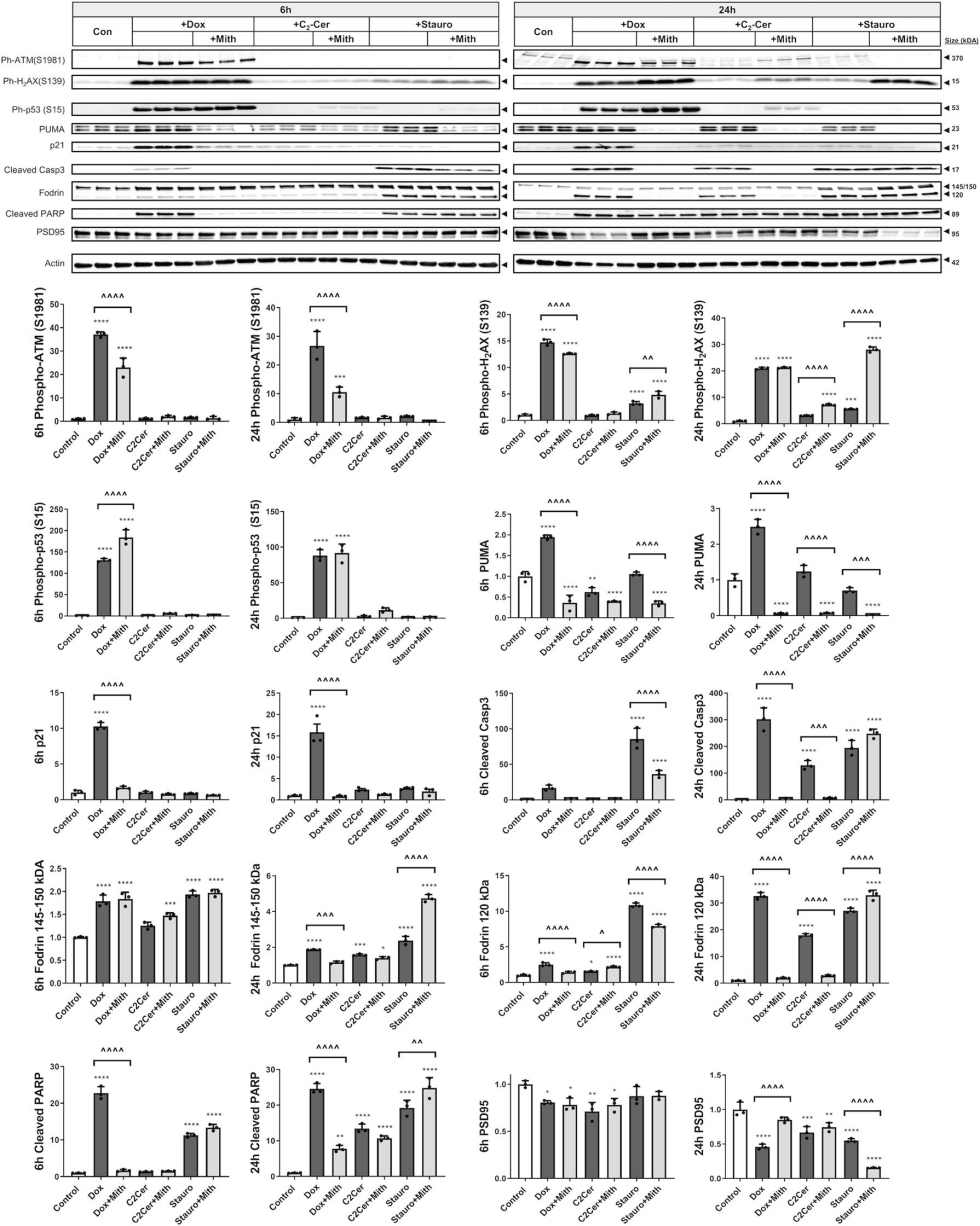
Mithramycin’s attenuation of apoptotic mechanisms in neurons is cell death model-specific. RCN were treated with 200 nM doxorubicin, 50 µM C_2_-ceramide, or 0.5 µM staurosporine +/−200 nM mithramycin. After 6 or 24 h, cells were harvested, equal amounts of whole-cell lysates were loaded onto an SDS-polyacrylamide gel and after electrophoretic separation and transfer to a membrane were incubated with antibodies against DNA-damage markers, phospho-p53 (S15) and its downstream proteins, PUMA and p21, as well as active caspase-3, its targets, PARP and Fodrin, and the synaptic marker PSD95. Protein levels (of bands indicated by arrows) were quantified by densitometry, normalized to β-actin signal and are presented as fold change compared with control levels. *n* = 3/groups for all groups. Data all represent mean ± SD. Significance assigned based on one-way ANOVA and Tukey post hoc test; *p < 0.05, **p < 0.01, ***p < 0.001, ****p < 0.0001 vs control; ^*p* < 0.05, ^^*p* < 0.01, ^^^*p* < 0.001, ^^^^*p* < 0.0001 vs cell death inducer alone.

To determine the range of mithramycin’s effects we examined its activity in various neuronal apoptosis models. Doxorubicin caused an early (6 h) and sustained (24 h) increased activation of DNA-damage responses including elevation of both phospho-ATM and γH_2_AX (western blot) (Fig. 5). In contrast, C_2_-ceramide and staurosporine did not significantly change phospho-ATM levels. Similarly, C_2_-ceramide had no effect on γH_2_AX levels while staurosporine only modestly increased γH2AX (Fig. 5). Mithramycin co-treatment robustly attenuated doxorubicin-induced phospho-ATM but only modestly attenuated γH2AX levels. Mithramycin co-treatment did not change phospho-ATM levels after C_2_-ceramide and staurosporine treatment and led to increased γH2AX levels. This latter effect was substantial after staurosporine 24 h treatment (Fig. 5).

Doxorubicin was the only inducer to significantly increase phospho-p53 (S15) levels and elevate PUMA and p21. Mithramycin co-treatment attenuated the doxorubicin-mediated changes downstream of phospho-p53 (S15). Consistent with changes observed in the etoposide model (Fig. 2), PUMA levels were lower in mithramycin co-treated samples in all three models (Fig. 5).

All three cell death models led to progressive caspase pathway activation, including early (6 h) elevation in cleaved Casp3 as well as increased caspase-dependent cleavage fragments of Fodrin (120 kDa) and PARP (89 kDa); these changes were further amplified at 24 h (western blot) (Fig. 5). The increase in the calpain/caspase-dependent cleavage fragments of Fodrin (145/150 kDa) was more modest. Mithramycin co-treatment consistently attenuated the doxorubicin and C_2_-ceramide caspase-dependent apoptotic markers but only temporarily (6 h) reduced several staurosporine-dependent changes, as well as causing an increase in staurosporine-dependent changes at 24 h. Consistent with the results of viability experiments (Fig. 1), mithramycin co-treatment attenuated the loss of neuronal marker PSD95 only in the doxorubicin paradigm and even accentuated late (24 h) PSD95 decline in the staurosporine model (Fig. 5).

**Fig. 6:**
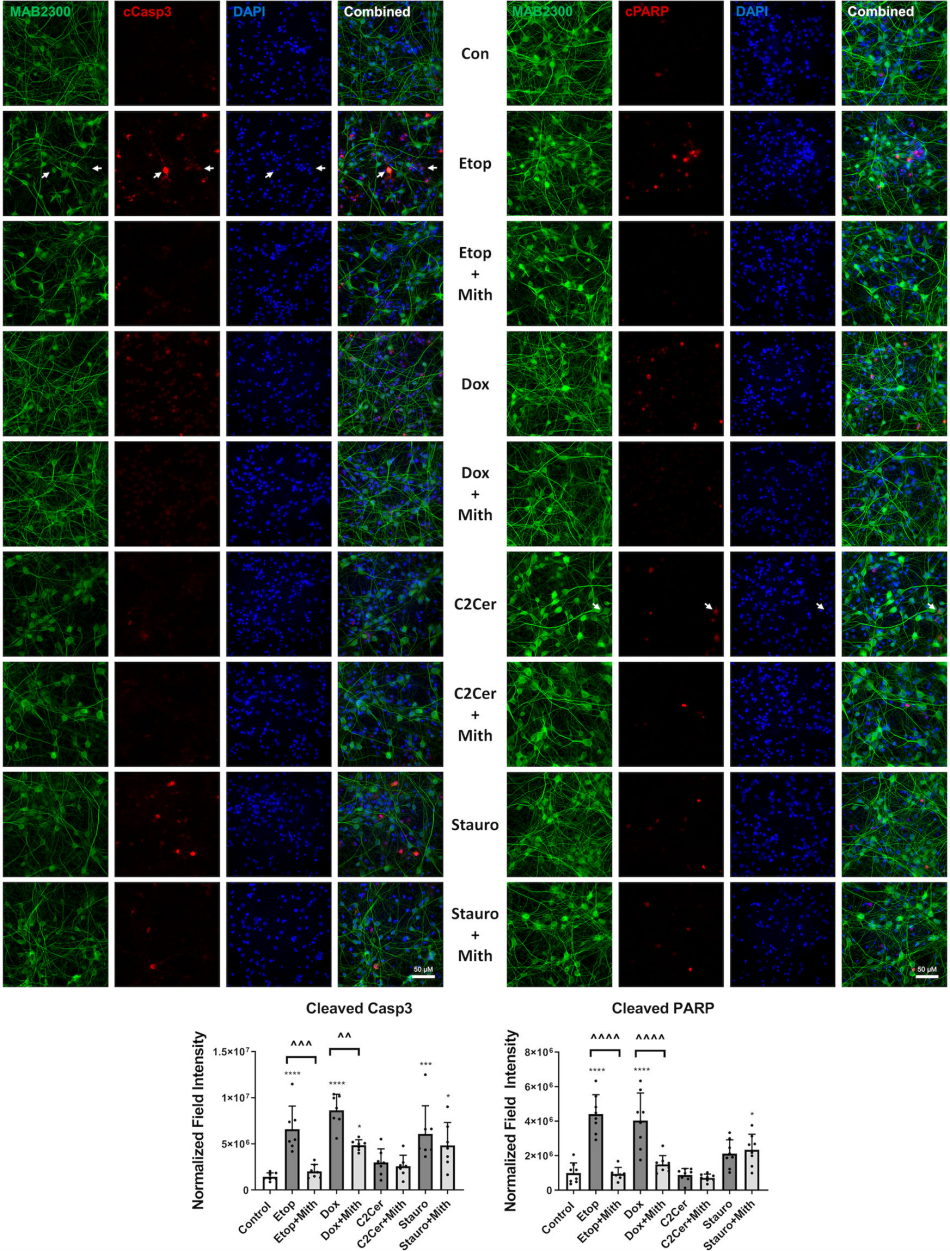
Mithramycin’s effect on immunofluorescent markers of caspase activation in neurons is cell death model-specific. RCN were treated with 200 nM doxorubicin, 50 µM etoposide, 50 μM C_2_-ceramide, or 0.5 μM staurosporine +/−200 nM mithramycin. After 6 h, cells were fixed and stained for neuronal markers (MAB2300, Neuro-Chrom™ Pan-Neuronal Marker), DAPI and cleaved Casp3 or cleaved PARP, imaged, and quantified. A representative field for each image at 63x magnification is shown. The Milli-Mark^TM^ Pan-Neuronal Marker is not able to successfully stain those neurons who are close to the end stages of cell death (caspase activation); however, white arrows indicate cleaved Casp3/cleaved PARP cells that show a clear neuronal morphology. Quantification was performed on at least seven fields for each treatment. Data all represent mean ± SD. Significance assigned based on one-way ANOVA and Tukey post hoc test; *p < 0.05, ***p < 0.001, ****p < 0.0001 vs control; ^^*p* < 0.01, ^^^*p* < 0.001, ^^^^*p* < 0.0001 vs cell death inducer alone.

We performed quantitative immunocytochemical analyses of activated caspase-3 and cleaved PARP at an early (6 h) time point to detect mithramycin-induced changes that mark the beginning of the execution phase of caspase-dependent neuronal death. Etoposide and doxorubicin led to significant increases in the number/intensity of cleaved caspase-3- and cleaved PARP-positive neurons while mithramycin co-treatment significantly attenuated these changes (Fig. 6). Consistent with western blot data (Fig. 5), C_2_-ceramide resulted in only modest changes. Staurosporine led to more substantial caspase-3 activation and PARP cleavage than C_2_-ceramide but these were not decreased by mithramycin co-treatment (Fig. 6).

### Mithramycin’s attenuation of the c-Jun injury response in neurons is cell death paradigm-specific

**Fig. 7:**
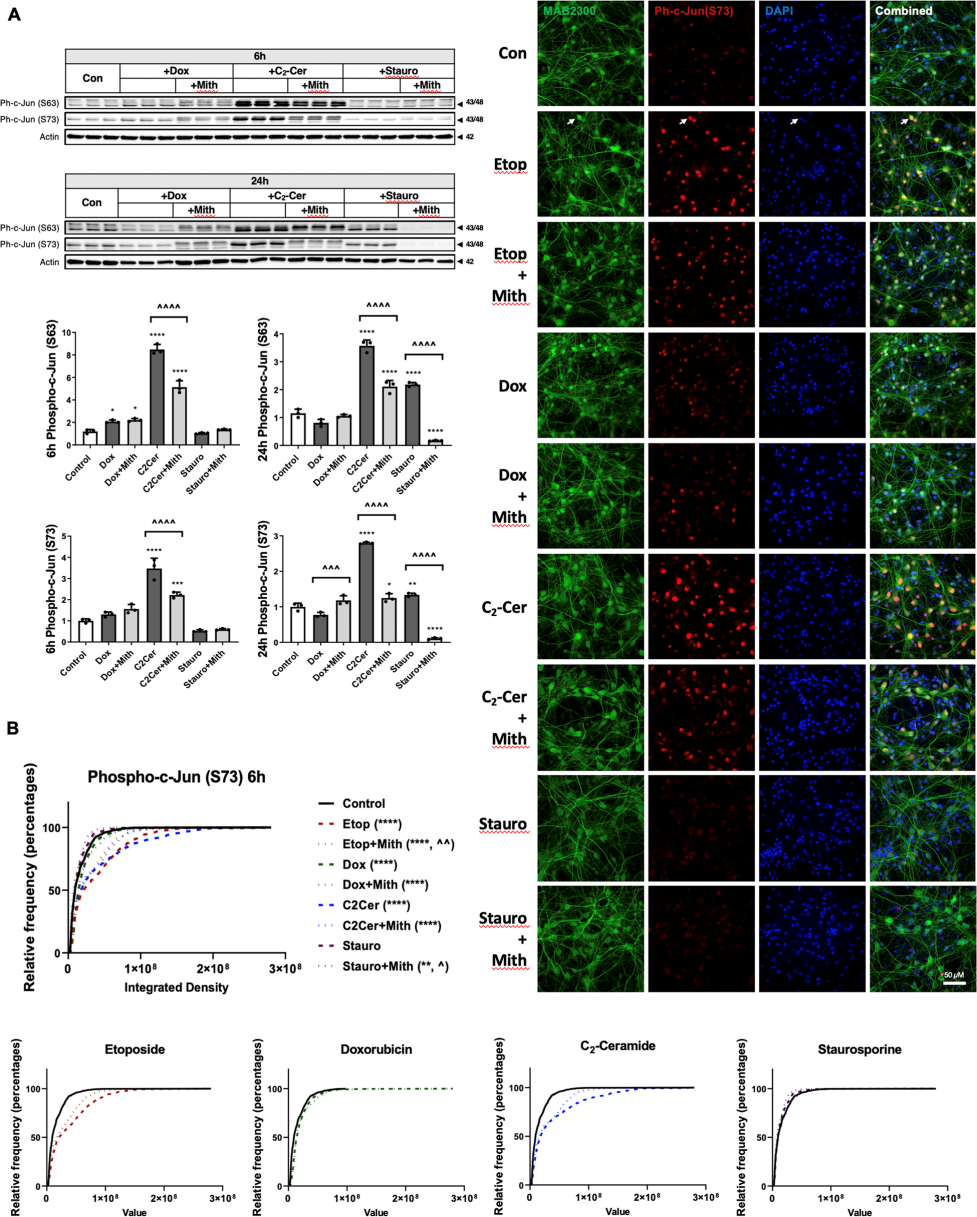
Mithramycin’s attenuation of the c-Jun injury reponse in neurons is cell death paradigm-specific. **a** RCN were treated with 200nM doxorubicin, 50 µM C_2_-ceramide, or 0.5 µM staurosporine +/−200 nM mithramycin. After 6 h or 24 h, cells were harvested, equal amounts of whole-cell lysates were loaded onto an SDS-polyacrylamide gel and after electrophoretic separation and transfer to a membrane were incubated with antibodies against phospho-c-Jun (S63) and phospho-c-Jun (S73). Protein levels (of bands indicated by arrows) were quantified by densitometry, normalized to appropriate β-actin signal and are presented as normalized fold change compared with control levels. Representative actin blots are shown here. *n* = 3/groups for all groups. Data all represent mean±SD. Significance assigned based on one-way ANOVA and Tukey post hoc test; *p < 0.05, **p < 0.01, ***p < 0.001, ****p < 0.0001 vs control; ^^^*p* < 0.001, ^^^^*p* < 0.0001 vs cell death inducer alone. **b** RCN were treated with 200nM doxorubicin, 50 µM etoposide, 50 µM C_2_-ceramide or 0.5 µM staurosporine +/−200 nM mithramycin. After 6h, cells were fixed and stained for neuronal markers (MAB2300, Neuro-Chrom™ Pan-Neuronal Marker), DAPI and phospho-c-Jun (S73), imaged and quantified. A representative field for each image is shown, white arrows indicate an example of a neuron co-localized with ph-c-Jun (S73). Quantification was performed on at least eight fields per treatment and is plotted as an unbinned cumulative frequency distribution. Significance assigned based on Kruskal–Wallis test and Dunn’s post hoc analysis; **p < 0.01, ****p < 0.0001 vs control; ^*p* < 0.05, ^^*p* < 0.01 vs cell death inducer alone.

We analyzed markers of c-Jun activation in various RCN apoptotic models using western blot for phospho-c-Jun (S73) and phospho-c-Jun (S63) and immunocytochemistry for phospho-c-Jun (S73). C_2_-ceramide resulted in rapid (6 h) c-Jun phosphorylation which remained at a high, albeit lowered, level at 24 h (western blot) (Fig. 7). Co-treatment with mithramycin robustly attenuated these changes. Doxorubicin caused only modest c-Jun activation at 6 h (S63) while staurosporine showed a late activation at 24 h (S63 and S73) (Fig. 7). Mithramycin co-treatment attenuated the latter changes. We used quantitative immunocytochemical analysis of phospho-c-Jun (S73) to characterize early-stage (6 h) neuronal c-Jun activation at single-cell resolution. We found that etoposide [mean rank diff. = 1339], C_2_-ceramide [mean rank diff. = 1213] and, to a far lesser extent, doxorubicin [mean rank diff. = 793.7] showed an increase in signal intensity of phospho-c-Jun (S73)-positive cells at 6 h, corresponding to a rightward shift of the population curve (Fig. 7). Mithramycin co-treatment attenuated staining intensity only in the etoposide group [mean rank diff. = 393.9], and had no significant overall effect on the doxorubicin group, possibly because the effect size of doxorubicin alone was very small, or on the C_2_-ceramide group, although it appeared to shift the distribution to the left but only in the range of the highest phospho-c-Jun (S73)-expressing cells (Fig. 7).

### Mithramycin treatment attenuates neuronal death pathways after experimental TBI in vivo

**Fig. 8:**
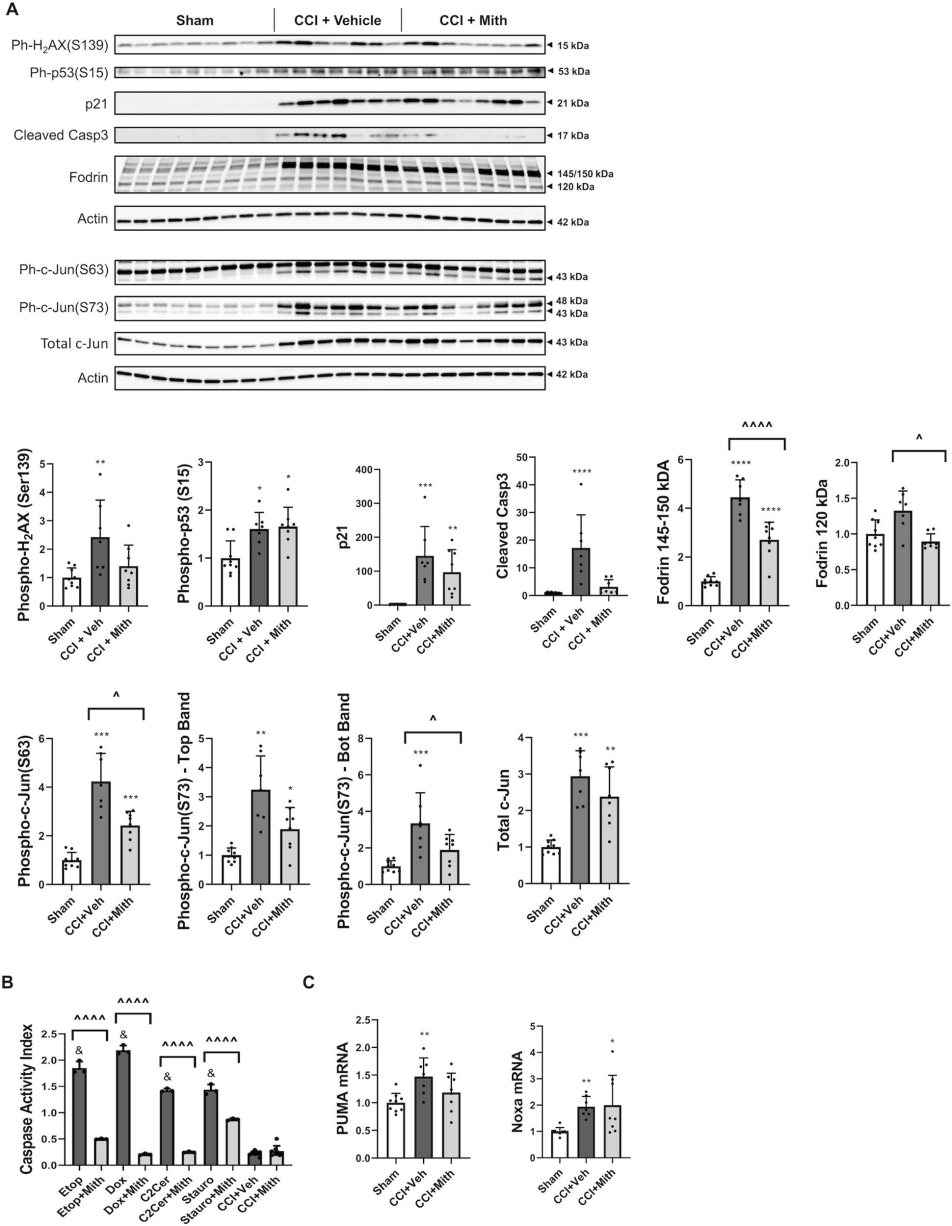
Mithramycin treatment attenuates neuronal death pathways after experimental TBI in vivo. **a** Whole-tissue lysates were obtained from ipsilateral-to-injury cortices 24 h after CCI+ intracerebroventricular injection of aCSF or mithramycin and from cortices of sham-injured animals. Equal amounts of whole-cell lysates were loaded onto an SDS-polyacrylamide gel and after electrophoretic separation and transfer to a membrane were incubated with antibodies against phospho-H_2_AX (S139), phospho-p53 (S15), p21, cleaved caspase-3, and Fodrin as well as against phospho-c-Jun(S63), phospho-c-Jun(S73), total c-Jun proteins. Protein levels (of bands indicated by arrows) were quantified by densitometry, normalized to appropriate β-actin signal and are presented as normalized fold change compared with sham-injured animals. Representative actin blots are shown here. **b** The Caspase Activity Index, or proportion of 120kDa to 145–150kDa Fodrin fragments was calculated for each cell death inducer or CCI +/− mithramycin at 24h. ^&^*p* < 0.0001 vs. CCI + Veh. **c** RNA was purified from the cortex and converted into cDNA. Equal volumes of cDNA were loaded for qPCR. mRNA levels were normalized via GAPDH, quantified using the 2^−ddCt^ method and are presented as fold change compared with control levels. *n* = 7+/group for all groups. Data represent mean±SD. Significance assigned based on one-way ANOVA and Tukey post hoc test, Brown–Forsythe ANOVA test followed by Dunnett’s T3 multiple comparisons test [ph-c-Jun (S63), ph-c-Jun (S73)-top band, total c-Jun, p21], or Kruskal–Wallis test followed by Dunn’s post hoc tests [ph-P53 (S15), p21, cleaved Casp3, Fodrin 120kDa]. *p < 0.05, **p < 0.01, ***p < 0.001, ****p < 0.0001 vs sham-injured animals; ^*p* < 0.05, ^^*p* < 0.01, ^^^*p* < 0.001, ^^^^*p* < 0.0001 vs CCI+Veh or inducer alone.

We examined the effects of mithramycin (icv administration) on neuronal cell death pathways’ activation 24 h after controlled cortical impact (CCI). CCI + Veh showed increased cortical levels of phospho-H_2_AX (S139), phospho-P53 (S15), p21, cleaved caspase-3, and Fodrin (145/150 kDa) compared with Sham. Mithramycin treatment significantly decreased levels of Fodrin cleavage (145/150 kDa and 120 kDa) compared with CCI + Veh. Although other markers’ mean values indicate mithramycin-induced attenuation of the injury-induced upregulation, these changes did not reach statistical significance due to the modest degree of the change and/or biological variability. Nonetheless, the absence of significant differences in cleaved caspase-3 between CCI + Mith and Sham groups suggests a mithramycin-dependent attenuation of injury upregulation of active caspase-3 (Fig. 8a). To comparably quantify effective caspase activity across models/treatments we defined the “Caspase Activity Index” based on substrate cleavage, as the ratio of 120 kDa/145–150 kDa Fodrin bands’ intensity. It inverted from a higher numerator in in vitro models, indicative of strong caspase activation, to a higher denominator after mithramycin co-treatment in vitro and after TBI in vivo. Mithramycin had no additional effect in vivo (Fig. 8b).

CCI + Veh also resulted in increased cortical levels of phospho-c-Jun (S63), phospho-c-Jun (S73), and total c-Jun compared with Sham mice. As the expected 48 kDa band of phospho-c-Jun (S63) was masked by a non-specific band, we separately quantified each c-Jun band. Mithramycin treatment significantly decreased phospho-c-Jun (S63 and S73, 43 kDa band) levels vs. vehicle; reductions in phospho-c-Jun(S73)’s 48 kDa band and total c-Jun did not reach significance (Fig. 8a).

CCI + vehicle administration also led to increases in cortical PUMA and Noxa mRNA (Fig. 8c). Mithramycin treatment did not significantly decrease either mRNA’s levels, although absence of significant differences in Puma between CCI + Mith and Sham may suggest a mithramycin-dependent attenuation of injury upregulation of PUMA.

## Discussion

Etoposide is a topoisomerase-II inhibitor that sequentially causes DNA strand breaks, p53 activation and intrinsic neuronal apoptosis^37,49,50^. Mithramycin reduced etoposide-induced neuronal death and improved long-term survival, suggesting that mithramycin causes a sustained transformation in the pattern/progression of apoptotic processes rather than a temporary delay.

Mithramycin was also neuroprotective in other DNA-damage models including exposure to camptothecin, a topoisomerase-I inhibitor^51^, or doxorubicin, a topoisomerase-II inhibitor, DNA intercalator and widely used chemotherapy agent^52^. In contrast, mithramycin was ineffective in staurosporine^53^ or C_2_-ceramide^54^ models of neuronal apoptosis, which are DNA-damage-independent^53,55^. Thus, mithramycin may preferentially target DNA-damage-dependent neuronal cell death pathways and its neuroprotective effects^30,33,56^ may be restricted to conditions where these mechanisms dominate.

Phosphorylation/activation of ATM is an early DNA-damage response, which serves to phosphorylate H_2_AX and initiate DNA repair pathways^57^ but may also activate kinases that stimulate p53 and downstream apoptosis pathways^58^. The balance between restorative and cell death programs determines neuronal fate after DNA damage^59^. Mithramycin neither inhibited etoposide-induced ATM and H_2_AX phosphorylation, nor reduced p53 phosphorylation, indicating that it does not directly affect early DNA damage/repair processes or p53 activation, but acts on downstream or independent apoptotic pathways. Mithramycin strongly attenuated etoposide-induced caspase-3/7 activation and decreased caspase substrate cleavage (PARP and Fodrin). The irreversible proteolytic cleavage of these substrates is a marker of entry into the execution phase of apoptosis, the end-stage for dying neurons^60^, while its attenuation is an oft-used marker of neuroprotection^61–63^. Upstream of caspase activation, MOMP has been identified as a critical and irreversible step in the intrinsic apoptosis pathway^64^. Etoposide treatment induced early (6 h) elevated cytosolic levels of AIF/CytC and a decline in mitochondrial respiration capacity/bioenergetic function; mithramycin attenuated these changes. Our results, confirmed in the doxorubicin model, indicate that mithramycin acts upstream of MOMP and caspase activation to protect neurons following DNA damage.

Phospho-p53 (S15) transactivates promoters of pro-apoptotic Bcl-2 family members leading to MOMP and apoptosis^65–67^. Mithramycin attenuated etoposide- and doxorubicin-mediated induction of key pro-apoptotic Bcl-2 family members, BH3-only molecules Puma and Noxa, as well as p21, a cell death modulator and well-known p53 target molecule^67^, despite no changes in p53 phosphorylation. Thus, Mithramycin regulates p53 pro-apoptotic transcriptional activity downstream of p53 phosphorylation across DNA-damage-dependent neuronal death models. Although we show broad Mithramycin-mediated down-regulation of injury markers, this is unlikely to be the result of a global non-specific decrease in transcription as mithramycin rescues levels of other mRNAs and proteins (e.g., PSD95) from etoposide-induced decline.

We have previously shown that miR-23a can target PUMA and Noxa mRNAs^35^. However, mithramycin did not attenuate the rapid etoposide-dependent miR-23a decline, indicating that its effects on Puma and Noxa are miR-23a-independent. Similarly, miR-711 is a pro-apoptotic microRNA induced by etoposide that targets the pro-survival molecules Ang-1 or Akt^49^ and can affect levels of Bcl-2 family members. As mithramycin co-treatment had no significant impact on these targets, except a late effect on Akt mRNA, Sp1 inhibition is unlikely to attenuate neuronal cell death by modulating the miR-711/PI3K/Akt pathway.

Sp1 has been shown to broadly alter p53’s ability to transactivate promoters and facilitate apoptosis induction^26,27,68^. Moreover, p53 and Sp family members interact in apoptotic gene regulation, and in some cell types, Sp1 is necessary for p53-mediated transcription of Bax and Puma, two Bcl-2 family members essential for p53-dependent apoptosis^28,69^. In our primary neuron ChIP experiments, Mithramycin inhibited Sp1 promoter binding without changing p53 binding to the same Noxa promoter region. Thus, our data show that mithramycin affects neither p53 phosphorylation not its recruitment to promoter sites and provide evidence that Sp1 is necessary for p53-mediated transcription of key pro-apoptotic molecules in DNA-damage-induced neuronal apoptosis, potentially by cooperating with p53 to transactivate pro-apoptotic gene promoters.

Unlike in DNA-damage-dependent models, we observed neither significant activation of p53-dependent apoptotic pathways, including p53 phosphorylation and BH3-only molecules’ induction, nor strong mithramycin neuroprotection in DNA-damage-independent models. Nonetheless, both C_2_-ceramide and staurosporine paradigms display robust caspase-3 substrate cleavage, suggesting that caspase pathway activation in those paradigms is significantly p53/BH3-independent. The involvement of model-specific neuronal death mechanisms is further evidenced by mithramycin-dependent attenuation of caspase activation after C_2_-ceramide but not after staurosporine. However, caspase activation does not appear necessary for C_2_-ceramide-induced neuronal death as mithramycin only modestly reduced the latter.

In concert with these changes in the canonical intrinsic apoptosis pathway, we also showed that mithramycin reduces etoposide-induced increases in protein levels of c-Jun (total and phosphorylated) and c-Fos as well as c-Jun mRNA levels. Modulation of c-Jun expression by mithramycin is likely due to the Sp1 sites present in the c-Jun promoter. Together, c-Jun and c-Fos dimerize to form the transcription factor AP-1^70^, which can cooperate with p53 and Sp1 to affect many genes, including pro-apoptotic Bcl-2 family members^71,72^. Our data therefore suggest that Sp1 modulation may affect two separate transcriptional pathways (p53 and AP-1) involved in neuronal cell death^73^. Moreover, as Sp1 has also been shown to cooperate with c-Jun to activate gene transcription^72,74^, mithramycin neuroprotective effects may also be due to directly modulating Jun-dependent transcription.

Although we show that induction of c-Jun pathways is a general neuronal injury response, its magnitude differs across models. Interestingly, it appears much weaker in doxorubicin vs. etoposide suggesting lack of uniform regulation even within DNA-damage models. In contrast, it is highly and rapidly upregulated by C_2_-ceramide, where mithramycin’s ability to suppress c-Jun levels supports a p53-independent mechanism. Despite the potential to disrupt c-Jun (and caspase) activation, mithramycin is not neuroprotective in the C_2_-ceramide model, suggesting these pathways are not among the dominant apoptosis-driving mechanisms in this model.

Using a mouse experimental TBI model (CCI), we detected acute DNA-damage responses^6^ and initiation of neuronal cell death mechanisms. Mithramycin modestly attenuated upregulation of some cell death pathways in the injured cortex 24 h after injury, including a significant reduction in levels of phosphorylated c-Jun (S63 and S73) and cleaved Fodrin fragments, and possible reduction in Puma and cleaved caspase-3. Brain Fodrin is primarily a neuronal protein^41^ whose cleavage is a neuronal death hallmark after acute brain injury^75,76^. Depending on the neuronal environment, Fodrin may be cleaved to calpain/caspase-generated 145/150 kDa fragments or to a caspase-generated 120 kDa fragment whose predominance reflects strong caspase activity^77^. Our previous studies showed repression of intrinsic caspase activation in the adult brain and low caspase activity acutely after experimental TBI^62,78^. Appropriately, here we detected only modest effective caspase activation, indicated by prevalence of the more calpain-specific 145/150 kDa Fodrin fragments, and a far lower Caspase Activity Index at 24 h post-injury, compared with in vitro models.

Hence, CCI in adult mice may trigger neuronal cell death phenotypes other than intrinsic apoptosis such as those involving calpains^78^. Our data show that mithramycin has more limited effects in calpain-dependent neuronal cell death including that induced by ceramide^79^. Thus, while mithramycin may still attenuate some p53-dependent intrinsic apoptotic program elements, including BH3-only molecules, downstream cell death proteases and caspase-independent mechanisms as well as c-Jun activation, the narrower nature of these effects and relatively modest role played by the caspase-dependent portion of this cascade will likely reduce the neuroprotective therapeutic effect of mithramycin in *acute* TBI. However, as mithramycin acts upstream of MOMP, it may reduce deleterious mitochondrial perturbations after TBI^80^, leading to long-term protection. Also, progressive de-repression of intrinsic caspase activation pathways may occur after the acute phase^62^, increasing mithramycin’s therapeutic window.

In summary, we have demonstrated profound neuroprotection by mithramycin in DNA-damage-induced and p53-dependent neuronal cell death following in vitro exposure to widely used chemotherapy agents. We have also shown that these protective effects are partially recapitulated acutely in an in vivo model of brain trauma, consistent with the more limited role played by p53-dependent intrinsic apoptosis. Thus, mithramycin and other interventions targeting Sp1 may provide avenues for novel neuroprotective strategies particularly in conditions where pathological changes are driven by activation of c-Jun or the p53-dependent intrinsic apoptosis pathway.
